# Guard Cell Metrics in Common and Japanese Hops from Wild, Feral, and Cultivated Backgrounds

**DOI:** 10.17912/micropub.biology.002068

**Published:** 2026-06-23

**Authors:** Adriana Seigneur, Puja Pradhan, Noah Williams, Geunhwa Jung

**Affiliations:** 1 Stockbridge School of Agriculture, UMass Amherst, Amherst Center, MA, US

## Abstract

Japanese hops are a closely related annual relative of the perennial crop species common hops, as such they are a potential genetic resource for accelerating hop breeding and cultivar development. Ploidy manipulation is an important tool in common hop breeding. In other crop systems, epidermal peels measuring guard cell traits-length, width, and plastid number-provide a rapid and low-cost method for screening ploidy levels. Guard cell length in Japanese hop exhibited intraindividual variability but appeared potentially less environmentally sensitive than plastid-based metrics in Japanese hop and trended higher in the tested triploid cultivar.

**Figure 1. Guard cell metrics of common and Japanese hops from wild, feral and cultivated backgrounds.** Number of plastids per guard cell pair (a), guard cell width (b), and guard cell length (c) in Japanese hop by sex and growing environment. The greenhouse female and greenhouse male groups each comprised five plants; the wild female group comprised seven plants and the wild male group three plants. Number of plastids per guard cell pair (d), guard cell width (e), and guard cell length (f) across hop groups of cultivated, feral, and wild origin. The diploid cultivar group comprised three plants, the feral group seven, and the Japanese hop group eleven. The seed-grown group comprised six plants; the *H. l. neomexicanus* and triploid cultivar groups were each represented by a single individual. Each data point represents the per plant mean of ten guard cell measurements.

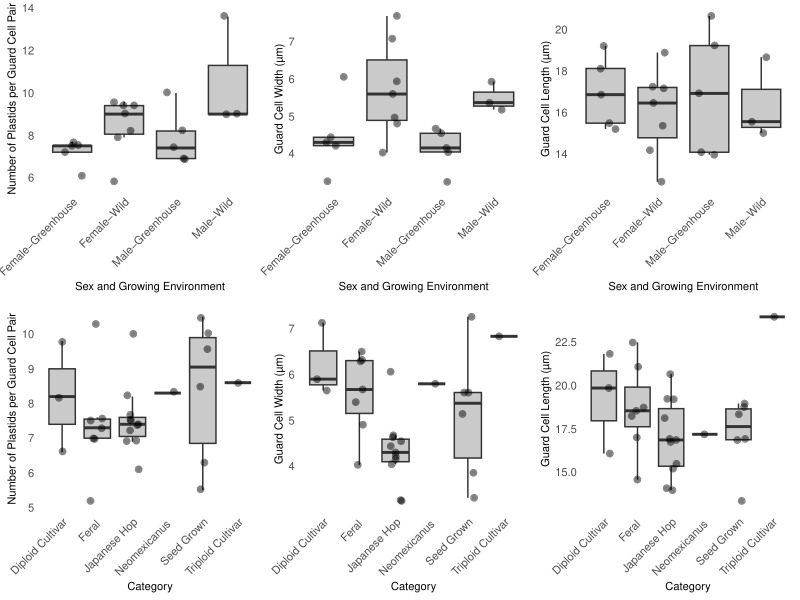



## Description

Common hop ( *Humulus lupulus* L.) and Japanese hop ( *Humulus scandens* (Lour.) Merr.; syn. *Humulus japonicus* Siebold & Zucc.) belong to the genus *Humulus* within the Cannabaceae family. Common hop is a dioecious, herbaceous perennial bine cultivated for its female inflorescences, which are economically important to the brewing and pharmaceutical industries (Paguet et al., 2023). Five botanical varieties of *H. lupulus* have been described: one native to Asia, one to Europe, and three to North America (Small, 1978). In the northeastern United States, native populations of *H. l.* var. *lupuloides* have interacted with feral hop plants that have persisted from abandoned farms since their introduction in the 1600s (McWilliams, 1998; Rumney, 1997, 1998; Somalraju et al., 2024). Many modern American cultivars contain genetic contributions from *H. l. lupuloides* and, more recently, *H. l. neomexicanus* (Small, 1978; Comi, 2024).

Japanese hop is an annual species native to East Asia and invasive in North America (Ehara, 1955; Small, 1978; Huang & Matthews, 2024). Unlike common hop, which produces economically important phytochemicals in lupulin glands, Japanese hop lacks lupulin glands and is grown primarily as an ornamental (Ehara, 1955, 1956; Paguet et al *.,* 2023). As a closely related annual relative of a perennial crop species, Japanese hop represents a potential genetic resource for accelerating hop breeding and cultivar development.

Ploidy manipulation has been widely used in crop improvement to enhance agronomic traits via polyploidization and to develop inbred lines through haploid induction in several crop systems, including potato, where these approaches involve multiple stages of ploidy screening (Hougas & Peloquin, 1957; Graebner, 2019; Williams et al., 2024). Common hop occurs primarily in the wild as a diploid (2n=20) with an XX/XY sex chromosome system. In contrast, Japanese hop exhibits 2n=16 in females and 2n=17 in males, with an XX/XY _1_ Y _2_ sex chromosome system (Zhang et al., 2023; Carey et al., 2024). Natural ploidy variation is present within the Cannabaceae family, including triploid species (Hayes et al., 2022). In common hop, aneuploids, triploids (3n), and tetraploids (4n) have arisen both spontaneously and through chemically induced polyploidization (Koutoulis et al., 2005). Triploids are particularly desirable due to their improved yield and production of nearly seedless cones, which are preferred by processors and brewers (Trojak-Goluch & Skomra, 2018, 2020).

Current hop breeding pipelines rely primarily on flow cytometry for ploidy determination, with leaf or root chromosome squashes used for cytological confirmation. Roy et al. (2001) reported average guard cell length and width for a limited sample of diploid and tetraploid common hops; however, comparative measurements for Japanese hops are not available (Šesek et al., 2000; Beatson et al., 2003; Trojak-Goluch & Skoma, 2020). In other crop systems, epidermal peels measuring guard cell traits-length, width, and plastid number-provide a rapid and low-cost method for screening ploidy levels (Frandsen, 1968). Guard cell size has also been correlated with DNA content and ploidy level in angiosperms fossil records, suggesting that size-based metrics may be broadly applicable across taxa, including *Humulus* (Masterson, 1994). The present study provides the first publicly available guard cell metrics for Japanese hops, *H. l. neomexicanus* , and feral hop populations from the northern United States. It also represents the first comparison of guard cell traits between greenhouse-grown and wild Japanese hop populations.

To assess intraindividual consistency of guard cell metrics, three sets of measurements from ten guard cells (length and width) or guard cell pairs (plastid count) were collected from a single greenhouse-grown Japanese hop (Swansea, MA). Guard cell length differed significantly among measurement sets (χ2=8.596, df=2, P=0.0136), whereas guard cell width (χ2=3.4692, df=2, P=0.1765) and plastid number (χ2=0.6286, df=2, P=0.7303) did not, indicating greater within-plant variability for length relative to the other traits. Plastid measurements were taken from greenhouse-grown Japanese hop plants and from a wild population, with each population further subdivided by sex. The greenhouse-grown group consisted of ten plants (five female, five male) collected from Wilmington, Concord, and Swansea, MA, while the wild group included ten plants (seven female, three male) identified on a slope in Amherst, MA and sampled in situ. For each individual, the mean of ten measurements was used as the value for each response variable (length, width, plastid count). Differences among the four groups (greenhouse female, greenhouse male, wild female, wild male) were evaluated using a Kruskal–Wallis test. Neither plastid count (χ²=6.537, df=3, P=0.0882), guard cell width (χ²=7.425, df=3, P=0.0595) or guard cell length (χ²=0.4939, df=3, P=0.9202) differed significantly among groups. Descriptive trends suggested lower plastid counts in greenhouse-grown females relative to wild plants and greater guard cell width in wild plants relative to greenhouse-grown plants, but these patterns were not statistically supported (Fig. 1a–c). 

Comparative analyses were conducted between greenhouse-grown Japanese hop and multiple common hop groups. The diploid cultivar group included cultivars 'UK Serebrianka', 'Tettnanger', and 'Petoskey'. The feral group was comprised of one individual collected from each of the following locations: Albany, NY; Troy, NY; Wales, MA; Sudbury, MA; Bedford, MA; Skowhegan, ME; and Southwest Harbor, ME. The Japanese hop group included the ten previously described plants as well as one unsexed plant grown from commercially available seed (Dee's Seeds, Port Saint Lucie, Florida). Data for *H. l. neomexicanus* were obtained from a single accession (PI 635445). The seed-grown common hop group consisted of six individuals: four derived from PI 689630 and two from commercially available seed (MySeedsCo, Oakland Gardens, NY). The triploid cultivar evaluated was 'Triple Pearle' (Somalraju et al., 2024). For each individual, the mean of ten measurements was used as the value for each response variable. Kruskal–Wallis tests detected a significant difference among groups for guard cell width (χ²=11.376, df=5, P=0.044), whereas plastid count (χ²=3.1895, df=5, P=0.6708) and guard cell length (χ²=5.953, df=5, P=0.3108) did not differ significantly among groups. A post-hoc Dunn's test with the Bonferroni correction revealed no significant pairwise difference among categories guard cell widths. Descriptive trends suggested that guard cell width tended to be greater in common hop than in Japanese hop groups, and that the triploid cultivar had longer guard cells than most other groups, but none of these patterns were statistically supported (Fig. 1d–f).

All guard cell metrics exhibited limitations when applied to the available germplasm. Epidermal peels are not typically effective for detecting single chromosome aneuploidy, and triploids can be difficult to distinguish from diploids using stomatal traits alone (Frandsen, 1968; Graebner, 2019). In the present study, genetic differences between the two Japanese hop populations are a confounding factor, as the genetic diversity and introduction history of Japanese hop populations in Massachusetts remain unknown. When using leaf peels for ploidy screening based on plastid measurements, environmental variation must also be considered. Environmental effects on stomatal traits have been documented in other crop systems and can be minimized by evaluating plants grown under uniform conditions (Frandsen, 1968). Consistent with this, guard cell width in Japanese hop showed a non-significant trend toward sensitivity to growing environment, though no pairwise differences were statistically supported after correction for multiple comparisons. Although guard cell length exhibited intraindividual variability, it appeared less environmentally sensitive than guard cell width in Japanese hop and showed a non-significant trend toward distinguishing the triploid cultivar from most other groups. Overall, this study establishes baseline guard cell measurements for Japanese hop and related Humulus germplasm, providing foundational data to support future ploidy manipulation and screening efforts. 

## Methods

**Site and Growing Conditions**

A population of Japanese hops was identified in Amherst, Massachusetts, consisting of approximately 20 individuals growing on a 60-degree slope. Field samples were collected between August and October 2025, with sampling completed prior to the first frost to avoid frost-induced physiological changes.

Vegetative-stage plants collected from Swansea, Concord, and Wilmington, Massachusetts, were transferred to the CNS Research Greenhouse at the University of Massachusetts, Amherst, MA. Plants were grown in 1- or 2.5-gallon containers filled with Pro-Mix BX growing medium and fertigated with a nutrient solution supplying 200 ppm nitrogen. Greenhouse environmental conditions were maintained at 70°F during the day and 65°F at night, with supplemental lighting provided to maintain a consistent 16-hour light/8-hour dark photoperiod.

**Sex Determination of Japanese Hop Plants**

Sex of Japanese hop plants was determined based on distinct reproductive morphological traits, using the same criteria for both field and greenhouse populations *.* Japanese hop is a dioecious species, with male and female flowers borne on separate individuals. Male plants produce branched panicles, whereas female plants develop cup-shaped perianths that form cone-like structures (Shepard et al., 2000, Ehara, 1956).

**Epidermal Peels**

Epidermal peels were obtained from one or two fully expanded mature leaves per sampled individual. Ten guard cell or guard cell pairs were measured, selection was arbitrary and performed by the worker who made the peel. If ten clear measurements could not be obtained from two or fewer leaves, the measurements were discarded and new peels were made. Peel and staining procedures were adapted from Ordoñez et al. (2017). The abaxial (underside) epidermis was carefully removed and mounted on a microscope slide containing two drops of Gram’s iodine solution. An additional two drops were applied to fully submerge the tissue, and a coverslip was placed over the sample, gently pressed to ensure even contact and remove air bubbles. Slides were stained for 10-30 minutes to achieve optimal contrast. Excess stain was removed by blotting the slide with a Kimwipe at the end of the staining period. 

**Imaging**

Samples were imaged using brightfield microscopy with an Axio Imager A2 at 40x magnification for stomatal measurements and 100x magnification when higher resolution was required to distinguish plastids. Images were captured using an Axiocam 503 Mono digital camera and analyzed with ZEN 2 (Blue Edition) software. Individual guard cell length and width were measured to the nearest 0.01 um, and plastid number was quantified per pair of guard cells following the approach described by Williams et al. (2024).

**Statistical Analysis**

Three sets of measurements of ten guard cells for length and width, and ten guard cell pairs for plastid count, from an arbitrarily selected Swansea Japanese hop plant were taken for each response variable to assess intraindividual variation. The non-parametric Kruskal–Wallis test was employed for each metric to determine whether the measurement sets differed significantly. For comparisons of Japanese hop guard cell metrics across four groups (greenhouse female, greenhouse male, wild female, wild male) and across hop categories (diploid cultivar, feral common hops, Japanese hops, *Humulus lupulus* var. *neomexicanus* , seed-grown, triploid cultivar), the mean of the ten measurements taken per individual was used as the value for each response variable; unequal group sizes increased heteroscedasticity. The Kruskal–Wallis test was therefore used for each response variable because it is relatively robust to heteroscedasticity. Where the Kruskal–Wallis test found significant differences, the post-hoc Dunn's test with Bonferroni correction was employed.

## Reagents

**Table:** 

Source	Reagent	Identifier
Hardy Diagnostics	Non-Stabilized Gram's Iodine	I008N

## Data Availability

Description: Raw data of guard cell measurements. . Resource Type: Dataset. DOI: https://doi.org/10.22002/qks9z-3p465
